# Modulation of Cancer-Associated Fibroblasts via the miR-624-5p/FAP Axis Drives Progression and Metastasis in Non-Small Cell Lung Cancer

**DOI:** 10.3390/cancers18020279

**Published:** 2026-01-16

**Authors:** Yan Zhao, Shuman Zhen, Xiaoxu Li, Xiaolin Chen, Xue Zhang, Xinming Zhao, Lihua Liu

**Affiliations:** 1 Department of Oncology, The Fourth Hospital of Hebei Medical University, Shijiazhuang 050011, China; yanzhao0928@hebmu.edu.cn (Y.Z.); zhangxuehappy@hebmu.edu.cn (X.Z.); 2 Hebei Provincial Key Laboratory of Tumor Microenvironment and Drug Resistance, Shijiazhuang 050011, China; chen201224@hebmu.edu.cn; 3 Department of Radiotherapy, The Fourth Hospital of Hebei Medical University, Shijiazhuang 050011, China; shumanzhen@hebmu.edu.cn (S.Z.); 48903528@hebmu.edu.cn (X.L.); 4 Department of Nuclear Medicine, The Fourth Hospital of Hebei Medical University, Shijiazhuang 050011, China; 5 Department of Tumor Immunotherapy, The Fourth Hospital of Hebei Medical University, Shijiazhuang 050035, China

**Keywords:** cancer associated fibroblasts (CAFs), ^68^Ga-FAPI-04 PET/CT, fibroblast activation protein (FAP), miR-624-5p, NSCLC, metastasis

## Abstract

Background: Cancer-associated fibroblasts (CAFs) are key mediators of metastatic progression in non-small cell lung cancer (NSCLC). Fibroblast activation protein (FAP) serves as the hallmark of CAF activation. However, the upstream regulation of FAP remains elusive, limiting stroma-targeted therapy development. Methods: ^68^Ga-FAP inhibitor (FAPI)-04 PET/CT imaging was performed on 61 NSCLC patients to evaluate the clinical significance of FAP. CAFs and normal fibroblasts (NFs) were isolated from patient tissues. Bioinformatic analysis and qRT-PCR were employed to screen and validate miRNAs. Functional assays (CCK-8, collagen contraction, wound healing, transwell co-culture) were utilized to investigate the role of miR-624-5p in regulating fibroblast activation and the effects on the metastatic potential of NSCLC cells. The targeting relationship between miR-624-5p and FAP was validated using FISH, dual-luciferase assay, and Western blotting. Results: ^68^Ga-FAPI-04 uptake was higher in advanced NSCLC (*p* < 0.001) and correlated with tumor size, lymph node metastases, and distant metastases (*p* < 0.05). Isolated primary CAFs significantly enhanced the migration and invasion of A549 and PC9 cells compared to NFs (*p* < 0.001). We identified miR-624-5p as a significantly downregulated miRNA in CAFs (*p* < 0.001). Functionally, miR-624-5p overexpression inhibited CAF proliferation and collagen contraction (*p* < 0.01) and reduced the proliferation, migration, and invasion capabilities of A549 and PC9 cells (*p* < 0.001). Mechanistically, miR-624-5p bound to FAP mRNA and negatively regulated FAP expression (*p* < 0.001), thus suppressing CAF activation and tumor metastasis. Conclusions: Our findings establish miR-624-5p as a novel upstream regulator that suppresses FAP expression, consequently inhibiting CAF activation and its pro-metastatic function. Targeting the miR-624-5p/FAP axis represents a promising therapeutic strategy for NSCLC metastasis.

## Simple Summary

In the tumor microenvironment, cancer-associated fibroblasts (CAFs) facilitate metastasis and contribute to the poor prognosis of non-small cell lung cancer (NSCLC). Fibroblast activation protein (FAP) serves as a key mediator of CAF activity, yet its upstream regulatory mechanisms remain unclear. In this study, we utilized ^68^Ga-FAP inhibitor (FAPI)-04 PET/CT imaging to visualize FAP expression in NSCLC patients, revealing its overexpression in advanced disease and correlation with metastatic progression. Using primary CAFs and normal fibroblasts isolated from human NSCLC tissues, we confirmed through functional assays that CAFs promote NSCLC metastasis. Importantly, through mechanistic investigation, we identified miR-624-5p as a significantly downregulated miRNA in CAFs. We further demonstrated that miR-624-5p directly targets FAP, thereby suppressing CAF activation and NSCLC metastasis. Our findings reveal a novel miR-624-5p/FAP regulatory axis in CAF activation and highlight its potential as a therapeutic target for restraining NSCLC progression.

## 1. Introduction

Metastasis is the leading cause of death in patients with non-small cell lung cancer (NSCLC) [1,2]. The dynamic tumor microenvironment (TME) plays a crucial role in facilitating this lethal process [3]. As a major stromal component, cancer-associated fibroblasts (CAFs) orchestrate multiple facets of malignancy, including tumor growth, invasion, and metastasis [4,5]. Notably, the persistent activation state of CAFs is critical for creating a permissive and invasive niche in the TME [6]. However, CAF activation is a complex biological process involving multiple factors, and its role and molecular mechanisms in NSCLC metastasis have not been fully elucidated.

Fibroblast activation protein (FAP), a type II transmembrane serine protease highly expressed on activated fibroblasts, is not only a marker that clinicians recognize but also a regulator of CAF activation [7]. Its abundance in the stroma strongly correlates with poor prognosis of NSCLC patients, underscoring its clinical significance [8,9]. FAP induces fibroblast activation to acquire a CAF phenotype, which facilitates tumor metastasis through dual mechanisms: it directly activates intracellular signaling pathways that upregulate genes promoting cell migration, including STAT3, PI3K/AKT, and RAS/ERK [10,11]. Moreover, FAP localizes to the invasive front of tumors and remodels the extracellular matrix, thereby indirectly fostering metastatic progression [12,13]. Despite its established pro-metastatic role, the upstream regulatory mechanisms of FAP expression and CAF activation remain elusive. This knowledge gap significantly hinders the development of stroma-targeted strategies.

Epigenetic modifications, such as noncoding RNA-mediated targeting, are significant hallmarks of cancer [14]. MicroRNAs (miRNAs) are highly conserved non-coding RNAs that can regulate target gene expression at the post-transcriptional level [15,16]. Aberrant expression of miRNAs in fibroblasts induces the transition of normal fibroblasts (NFs) to the CAF phenotype, thereby enhancing cancer-promoting capabilities [17,18,19]. Studies have shown that miR-624-5p is associated with cancer cell migration and invasion [20,21], but the biological function of miR-624-5p in CAFs and NSCLC remains unclear. This study aims to incorporate FAP-targeted molecular imaging to characterize CAF activity in NSCLC patients using ^68^Ga-FAPI-04 PET/CT and to investigate the epigenetic regulation of FAP, with a particular focus on microRNAs as upstream modulators. By integrating molecular imaging with mechanistic validation, this study provides evidence for developing therapeutic strategies targeting CAFs to complement conventional tumor cell-directed treatments.

## 2. Materials and Methods

### 2.1. ^68^Ga (Gallium)-FAP Inhibitor (FAPI) PET/CT Imaging of NSCLC Patients

Between February 2022 and June 2024, 61 patients with a confirmed diagnosis of NSCLC received ^68^Ga-FAPI-04 PET/CT at our institution. The study protocol was approved by the Hospital Ethics Committee (Approval No. 2021069). Imaging was carried out using a Dutch Philips Vereos fully digital PET/CT system. The precursor FAPI-04 was provided by TZ-Bio (Nanchang, China), and the radiotracer ^68^Ga-FAPI-04 (radiochemical purity > 95%) was injected intravenously at 1.8–2.2 MBq/kg body weight. After a 60 ± 5-min uptake period, whole-body acquisition from the head to the upper thighs was performed. CT parameters were set at 120 kV and 60 mA, with a 4 mm slice thickness for attenuation correction. PET images were acquired in 3D mode using a 512 × 512 matrix, 4 mm slice thickness, and an acquisition time of 2 min per bed position, covering 8–10 bed positions in total. The CT data were utilized to correct the attenuation of the PET images, which were then fused with the CT images. Visual and semi-quantitative assessments were performed on maximum intensity projection (MIP) and sectional images to determine the maximum standardized uptake value (SUVmax) and mean standardized uptake value (SUVmean) in the tumor lesion. The expression of FAP in the primary pulmonary lesions of the patients and the status of metastasis were observed.

### 2.2. Cell Lines

Human lung adenocarcinoma cell lines A549 and PC9 were purchased from Procell Life Science & Technology Co., Ltd. (Wuhan, China). The cells were cultured in RPMI-1640 medium (Gibco, Waltham, MA, USA) supplemented with 10% FBS, 100 U/mL penicillin, and 100 µg/mL streptomycin at 37 °C with 5% CO_2_.

### 2.3. Fibroblast Isolation and Culture of NSCLC Patients

Isolation of CAFs and NFs was performed using tumor and distal para-cancerous (>5 cm) tissues from five NSCLC patients, under the approved ethics protocol of our hospital (No. 2020KY166). Post-operative tissues were promptly washed in PBS with antibiotics (100 U/mL penicillin, 100 µg/mL streptomycin), minced, and digested for 2 h at 37 °C using a solution of DNase (2 mg/mL, Gibco, Waltham, MA, USA) and collagenase type I (5 mg/mL, Gibco, Waltham, MA, USA) in the presence of the antibiotics. Following filtration and centrifugation (800 r/min, 8 min), cells were cultured in DMEM-based medium containing 15% FBS and 20 ng/mL epidermal growth factor (PeproTech, Rocky Hill, NJ, USA). The suspended cells were inoculated in cell culture flasks and cultured in an incubator at 37 °C with 5% CO_2_. Fibroblasts were subcultured when they reached 80% confluence. The fibroblasts from passages 3 to 8 were used for subsequent experiments.

### 2.4. Cell Immunofluorescence (IF) and FISH

For IF staining, fibroblasts were identified using markers for FAP, α-smooth muscle actin (α-SMA), and the epithelial cell marker epithelial cell adhesion molecule (EpCAM). Cells were fixed with 4% paraformaldehyde for 30 min, followed by PBS washing, permeabilization with 0.5% Triton X-100 (Solarbio, Beijing, China), and blocking with 10% BSA, and then incubated overnight at 4 °C with antibodies against FAP (Bs-5758R, 1:100, Bioss, Woburn, MA, USA), α-SMA (ab124964, 1:100, Abcam, Cambridge, MA, USA), and EpCAM (ab71916, 1:100, Proteintech, Chicago, IL, USA). Subsequently, they were treated with goat anti-rabbit secondary antibody (ABclonal, Wuhan, China) at 37 °C for 1 h. The cell nuclei were counterstained with DAPI (Solarbio, Beijing, China). For the FISH assay, the Dig-labeled miR-624-5p probe was designed and synthesized by Servicebio (Wuhan, China). The probe sequence was as follows: 5′-TGAACACAAGGTACTGGTACTA-3′. Hybridization was carried out overnight following the manufacturer’s protocol, and images were captured using a fluorescence microscope (Nikon, Tokyo, Japan).

### 2.5. Bioinformatic Analysis

Microarray data profile GSE169587 was retrieved from the Gene Expression Omnibus (GEO) database to interrogate miRNA expression differences between lung cancer tissues and matched adjacent normal tissues. Differentially expressed miRNAs meeting the criteria of |fold change| ≥ 2 and *p* value < 0.05 were deemed statistically significant and selected for subsequent analysis by R software V4.2.0.

### 2.6. Quantitative Real-Time Polymerase Chain Reaction (qRT-PCR) Assay

In brief, miRNA was isolated following the instructions of the MiPure Cell/Tissue miRNA Kit (Vazyme, Nanjing, China). 1 μg of extracted miRNA was used as a template for reverse transcription into cDNA for qPCR amplification. The reaction conditions were set as follows: 95 °C for 5 min, 95 °C for 10 s, and 60 °C for 30 s for 40 cycles. The primers were synthesized by RiboBio (Guangzhou, China). miR-624-5p forward primer: 5′-ACACTCCAGCTGGGGTAGTACCAGTACCTTG-3′, miR-624-5p reverse primer: 5′-TGGTGTCGTGGGAGTCG-3′; U6 forward primer: 5′-CTCGCTTCGGCAGCACA-3′, U6 reverse primer: 5′-AACGCTTCACGAATTCG-3′. U6 was utilized as an internal reference, and the relative expression level of miR-624-5p was calculated using the 2^−ΔΔCT^ method.

### 2.7. miRNA Mimic and Inhibitor Transfection

CAFs were inoculated in 6-well plates when growth fusion reached 60% to 70%. The miR-624-5p mimic, miR-624-5p inhibitor, and miRNA mimic negative control (NC), and miRNA inhibitor NC (RiboBio, Guangzhou, China) were transfected into fibroblasts using Lipofectamine 3000 following the manufacturer’s instructions.

### 2.8. Cell Proliferation Assay

Cell proliferation was evaluated using both the CCK-8 and colony formation assays. For the CCK-8 assay, 1 × 10^3^ cells were seeded per well in 96-well plates. At 0, 24, 48, and 72 h, 10 μL of CCK-8 reagent (Servicebio, Wuhan, China) was supplemented to each well. After a 2 h incubation, the absorbance at 450 nm was recorded using a microplate reader (BioTek Instruments, Winooski, VT, USA). The colony formation assay was performed by plating 800 cells per well in 6-well plates and culturing them in fibroblast-conditioned medium for two weeks. After fixation with 4% paraformaldehyde and staining with crystal violet, colonies containing ≥50 cells were counted.

### 2.9. Collagen Contraction Assay

Fibroblasts (5 × 10^5^ cells/mL) were suspended and mixed with Rat Tail Collagen I (Corning Incorporated, Corning, NY, USA) at a final concentration of 2 mg/mL. 500 μL of the mixture was added to the 24-well plates and allowed to solidify at 37 °C for 30 min. After the mixture solidified, a 20 μL sterile tip was used to separate the solidified collagen from the plate. Then, 1 mL of DMEM with 10% FBS was added to each well and incubated for 12 h. Changes in the collagen area were observed and measured. The collagen contractility was calculated as follows: Area (well) − Area (gel)/Area (well).

### 2.10. Conditioned Medium (CM) for Fibroblasts and Coculture of CAFs and Cancer Cells

Fibroblasts were grown to 80% confluence, washed twice with PBS, before adding serum-free DMEM. After incubating for 48 h at 37 °C with 5% CO_2_, the cell supernatant was collected and centrifuged (3000 r/min, 10 min, 4 °C). The CM was either analyzed cytologically or stored at −80 °C. In coculture setups, CAFs were placed in the lower compartment of a Corning (Corning, NY, USA) transwell insert (0.4 μm), while A549 or PC9 cells were grown in the insert of the upper chamber.

### 2.11. Cell Migration and Invasion Assay

For the migration assay, A549 and PC9 cells were inoculated in 6-well plates and scratched with a 200 μL sterile tip when they reached 90% confluence. Then, 2 mL of the indicated fibroblast CM was added. The migration of the cells was observed under a microscope at 0 and 24 h, and the migration distance toward the scratched area was measured. For the invasion assay, 2.5 × 10^4^ A549 cells or PC9 cells were suspended in 200 μL of serum-free 1640 medium and inoculated into the upper chamber of the Transwell plates, which are coated with Matrigel gel (diluted 1:8). The lower chamber was filled with fibroblast CM with 20% FBS. After 24 h of incubation (37 °C, 5% CO_2_), the upper chambers were gently washed to remove the Matrigel and any non-invading cells. The remaining cells were fixed and stained using 4% paraformaldehyde and 1% crystal violet, respectively. Invading cells were quantified by counting five random fields per well under a microscope at 200× magnification.

### 2.12. Dual-Luciferase Reporter Assay

The target genes of miR-624-5p were predicted by the TargetScan database, and the complementary binding sites of miR-624-5p and the 3′-untranslated regions (3′UTR) of FAP were investigated. Wild-type (WT-FAP 3′UTR) and mutant (MUT-FAP 3′UTR) luciferase reporter vectors, containing FAP 3′ UTR, were constructed by GenePharma (Shanghai, China). These vectors were subsequently co-transfected with either miR-624-5p mimic or miR-NC into CAFs via LipofectamineTM 3000 (Thermo Fisher Scientific, Waltham, MA, USA), and were cultured for 48 h. The luciferase reporter gene kit (Promega, Madison, WI, USA) was used to detect luciferase activity according to the manufacturer’s instructions.

### 2.13. Western Blotting Analysis

The BCA protein assay kit (Report Biotechnology, Xiamen, China) was used to quantify the total protein. 40 μg of protein from each sample was boiled for 5 min and then cooled, subjected to SDS-PAGE, and transferred to a PVDF membrane (Millipore, Billerica, MA, USA). Membranes were blocked with 5% bovine serum albumin for 2 h at room temperature, incubated overnight at 4 °C with FAP antibody (ab207178, 1:1000, Abcam, Cambridge, MA, USA). The membrane was washed with TBST, after which HRP-conjugated secondary antibodies (ABclonal, Wuhan, China) were added and incubated for 2 h. The protein bands were visualized by ECL (GE, Boston, MA, USA) on an Epson Perfection V39 scanner.

### 2.14. Statistical Analyses

Statistical analyses were performed using IBM SPSS Statistics 26.0 (IBM, Armonk, NY, USA) and GraphPad Prism 9.2 (GraphPad, La Jolla, CA, USA). All measurement data are presented as the mean ± standard deviation (SD). Differences between the two groups were analyzed by *t*-tests, while differences among multiple groups were assessed by the Kruskal–Wallis test. A *p*-value of less than 0.05 was considered statistically significant.

## 3. Results

### 3.1. ^68^Ga-FAPI-04 PET/CT Imaging of NSCLC Patients Demonstrates That FAP Is Associated with Tumor Progression and Metastasis

**Figure 1:**
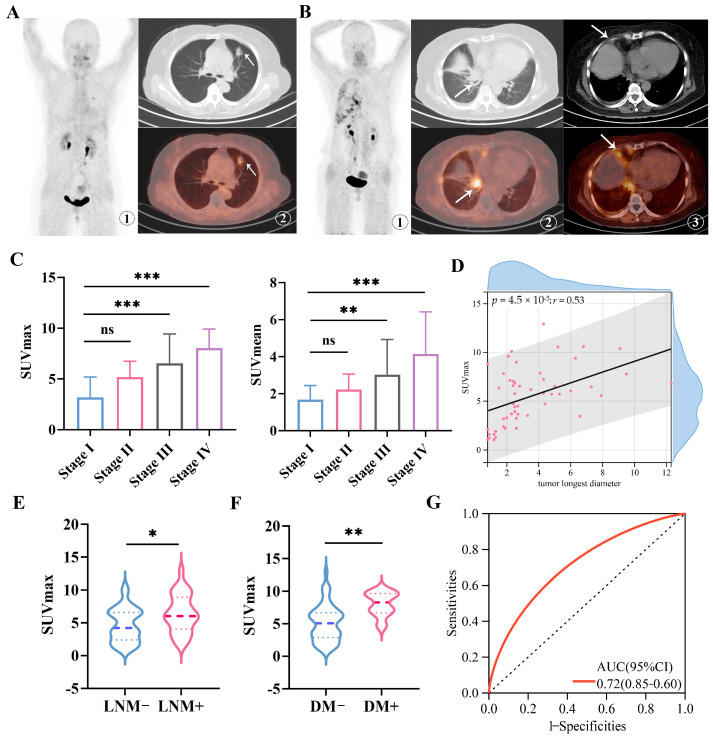
Imaging of ^68^Ga-FAPI-04 PET/CT in primary lesions of early and advanced NSCLC patients. (**A**) Representative ^68^Ga-FAPI-04 PET/CT imaging of pulmonary lesions in a patient with left lung adenocarcinoma (female, 64 years old, T1bN0M0, Stage Ia) (lesions indicated by arrows). ①: MIP image of ^68^Ga-FAPI-04; ②: CT image and the corresponding ^68^Ga-FAPI-04 fusion image, with SUVmax of 3.3; (**B**) Representative ^68^Ga-FAPI-04 PET/CT imaging of pulmonary lesions in a patient with right lung adenocarcinoma (female, 62 years old, T1cN0M1a, Stage IVa) (lesions indicated by arrows). ①: MIP image of ^68^Ga-FAPI-04; ②: CT image of the primary lung lesion and the corresponding ^68^Ga-FAPI-04 fusion image, with SUVmax of 9.6; ③: CT image of pleural metastatic lesion and the corresponding ^68^Ga-FAPI-04 image, with SUVmax of 5.1; (**C**) SUVmax and SUVmean values of NSCLC primary lesions in different TNM stages. (**D**) Correlations between the SUVmax of ^68^Ga-FAPI-04 and the tumor’s longest diameter. (**E**) SUVmax values of NSCLC primary lesions in patients with or without lymph node metastasis. (**F**) SUVmax values of NSCLC primary lesions in patients with or without distant metastasis. (**G**) The receiver operating characteristic (ROC) curve for SUVmax of ^68^Ga-FAPI-04 in predicting metastasis status in the clinical cohort. The area under the curve (AUC) is 0.72 (95% CI, 0.60–0.85). LNM, lymph node metastasis; DM, distant metastasis. ns: not significant, * *p* < 0.05, ** *p* < 0.01, *** *p* < 0.001. *r* = 0.53.

The study included 61 patients diagnosed with NSCLC, comprising 36 males and 25 females, with an average age of (65.18 ± 8.18) years. The clinical characteristics of the patients are summarized in Table 1. Representative ^68^Ga-FAPI-04 PET/CT imaging with early-stage lung adenocarcinoma (stage Ia, T1bN0M0) revealed a hyperdense nodule in the left upper lobe measuring about 2.0 cm × 1.3 cm. The scan showed low FAPI-04 uptake, with a SUVmax of 3.3 (Figure 1A). In contrast, representative ^68^Ga-FAPI-04 PET/CT imaging with advanced pulmonary adenocarcinoma at stage IVa (T1cN0M1a) exhibited a 2.1 cm × 1.7 cm lesion in the right hilum with intense FAPI-04 uptake (SUVmax 9.6). Additionally, high uptake of ^68^Ga-FAPI-04 was also observed in the right pleura (SUVmax 5.1) (Figure 1B).

The PET imaging analysis revealed significantly higher uptake of ^68^Ga-FAPI-04 (as measured by both SUVmax and SUVmean) in patients with advanced-stage (stage III–IV) NSCLC compared to those with early-stage (stage I–II) disease (both *p* < 0.001; Figure 1C). Furthermore, ^68^Ga-FAPI-04 SUVmax was positively correlated with tumor longest diameter, suggesting an association between FAP expression and tumor burden (Spearman’s rho = 0.53, *p* < 0.001; Figure 1D). Notably, patients without lymph node or distant metastases exhibited significantly lower tracer uptake than those with metastatic involvement (lymph node metastasis: *p* < 0.05, Figure 1E; distant metastasis: *p* < 0.01, Figure 1F). In addition, receiver operating characteristic (ROC) analysis indicated that ^68^Ga-FAPI-04 SUVmax could discriminate non-metastatic NSCLC from metastatic NSCLC with an area under the curve (AUC) of 0.72 (95% CI: 0.60–0.85; Figure 1G). Collectively, these findings demonstrate that ^68^Ga-FAPI-04 PET imaging robustly reflects in vivo FAP expression in NSCLC, which is upregulated in advanced disease and associated with metastatic progression. Our results support the potential clinical utility of ^68^Ga-FAPI-04 as an imaging biomarker for staging and prognostic assessment. Further investigation is warranted to elucidate the molecular mechanisms underlying CAF activation and FAP expression in NSCLC.

### 3.2. Isolation and Identification of CAFs and NFs in NSCLC Patients

**Figure 2:**
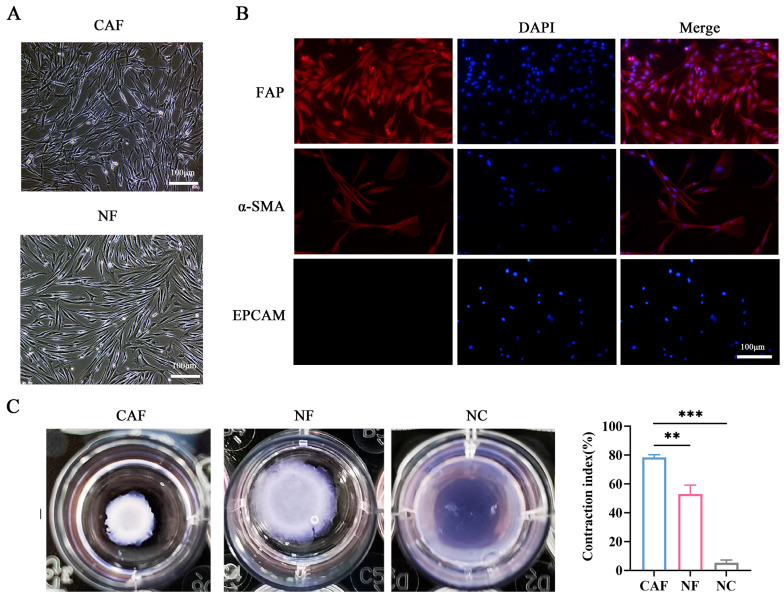
Isolation and identification of primary CAFs and NFs from NSCLC patients. (**A**) Morphological observation of CAFs and NFs under light microscopy (scale bar, 100 μm). (**B**) Immunofluorescence staining of FAP (red), α-SMA (red), and EpCAM (epithelial marker) in CAFs (scale bar = 100 μm), the nucleus (blue) was stained with DAPI. (**C**) Collagen contraction assay to detect the contraction ability of CAFs and NFs. ** *p* < 0.01, *** *p* < 0.001.

To further explore the molecular mechanisms of CAF activation and FAP expression, we isolated CAFs and normal fibroblasts (NFs) from tumor tissues and adjacent normal tissues (*n* = 5). The characteristics of fibroblasts were further identified by morphology and immunofluorescence (Figure 2A,B). The immunofluorescence staining indicated that CAFs were positive for both α-SMA and FAP, while they did not express EpCAM (Figure 2B). Additionally, the collagen contraction assay was performed to assess the contraction ability of CAFs and NFs. CAFs demonstrated considerably higher contraction capability than NFs, indicating the active state of fibroblasts (*p* < 0.01, Figure 2C). Overall, we successfully isolated and cultured CAFs and NFs from NSCLC tissue.

### 3.3. CAFs Promote the Migration and Invasion of A549 and PC9 Cells

**Figure 3:**
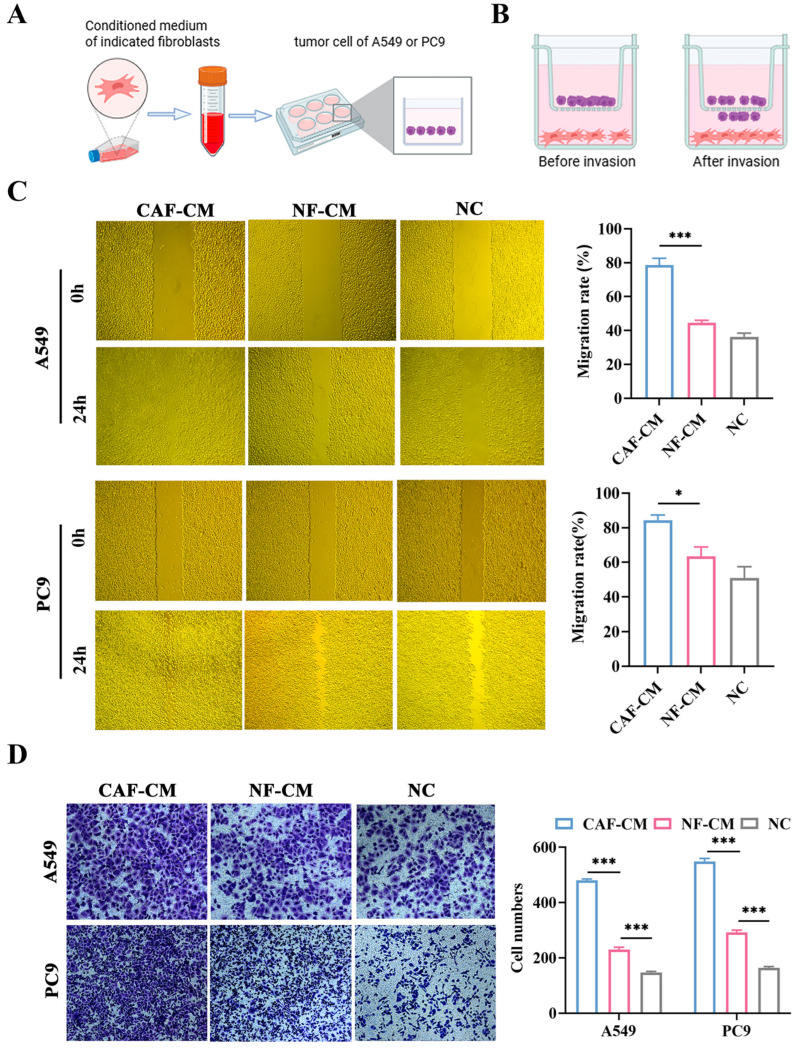
The impact of CAFs and NFs on the migration and invasion abilities of A549 and PC9 cells. (**A**) Schematic diagram showing that the conditioned medium of fibroblasts was added to NSCLC cells for subsequent experiments; created in BioRender. Shuman Zhen. https://www.biorender.com/mbog627 (accessed on 6 January 2026). (**B**) Schematic diagram of the transwell coculture system, created in BioRender. Shuman Zhen. https://www.bioRender.com/penmd3c (accessed on 6 January 2026). (**C**) Scratch assay to assess the effect of CAF-CM and NF-CM on the migration ability of A549 and PC9 cells (×200). (**D**) Transwell coculture assay to assess the effect of CAFs and NFs on the invasion ability of A549 and PC9 cells, crystal violet staining (×200). * *p* < 0.05, *** *p* < 0.001.

To evaluate the impact of CAFs and NFs on the ability of NSCLC cells to migrate and invade, we collected conditioned media from CAFs (CAF-CM) and NFs (NF-CM) and established the transwell coculture system of fibroblasts and cancer cells (Figure 3A,B). The wound healing assays showed that compared to the NF-CM and NC groups, the CAF-CM group significantly increased the migration of A549 and PC9 cells (*p* < 0.001 and *p* < 0.05, respectively, Figure 3C). Additionally, the transwell coculture system indicated that CAFs enhanced the invasion of A549 and PC9 cells (both *p* < 0.001, Figure 3D). These findings demonstrated that CAFs promote A549 and PC9 cell migration and invasion more than NFs.

### 3.4. miR-624-5p Is Downregulated in CAFs of NSCLC and Inhibits CAF Activation

**Figure 4:**
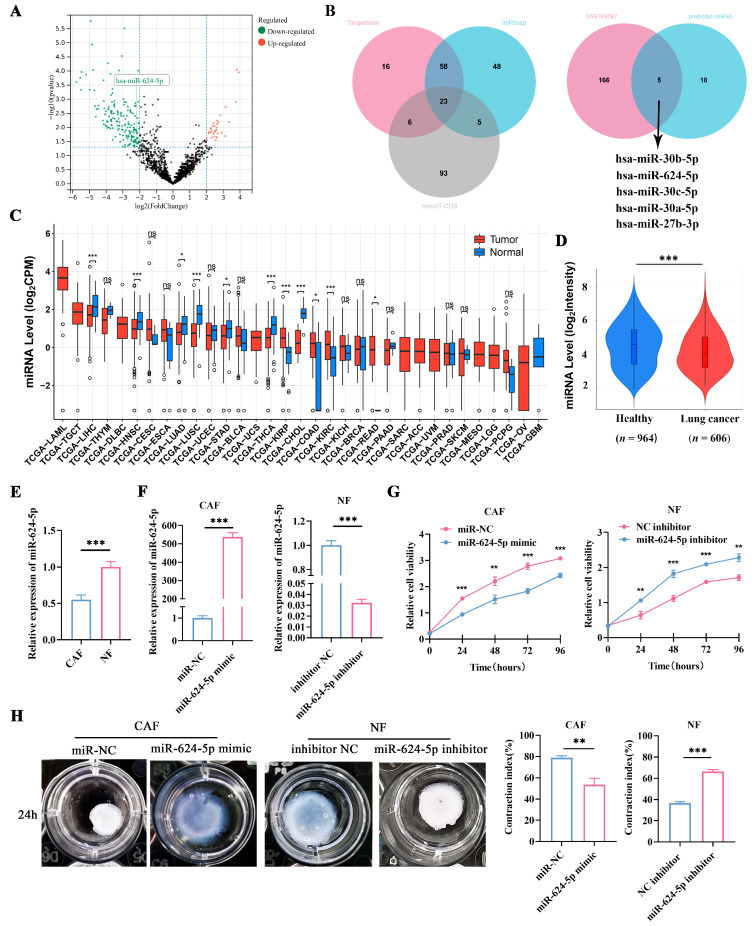
Expression of miR-624-5p in NSCLC and its effect on proliferation and activation of fibroblasts. (**A**) Volcano plot revealed differentially expressed miRNAs between NSCLC tissues and normal lung tissues in GSE169587. (**B**) Venn analysis of the potential binding miRNAs of FAP, predicted by TargetScan, miRmap, and microT-CDS, and overlapping miRNAs of GSE169587 and potential binding miRNAs. (**C**) Bioinformatics analysis of miR-624-5p expression in tumor tissues using the CancerMIRNome database. (**D**) Expression levels of miR-624-5p in the blood of healthy individuals and lung cancer patients using the E-MTAB-8026 database. (**E**) Expression levels of miR-624-5p in primary CAFs and paired NFs from NSCLC patients. (**F**) miR-624-5p expression after overexpression and knockdown of miR-624-5p was detected by qRT-PCR. (**G**) CCK-8 assay to detect the effect of miR-624-5p on the proliferation of CAFs and NFs. (**H**) Collagen contraction assay to evaluate the effect of miR-624-5p on the collagen contraction ability of CAFs and NFs. ns: not significant, * *p* < 0.05, ** *p* < 0.01, *** *p* < 0.001.

Accumulating evidence highlights the critical role of miRNA-mRNA interactions in cancer biology [22,23]. To investigate the mechanism by which CAFs promote the metastasis and progression of NSCLC, we analyzed the GEO dataset GSE169587 to identify dysregulated miRNAs in lung cancer tissues. Using a fold-change threshold of 2, we focused on significantly downregulated miRNAs (Figure 4A). Subsequently, we employed three bioinformatic algorithms (TargetScan, microT, and miRmap) to predict miRNAs potentially targeting FAP, which yielded 23 candidates (Figure 4B). A Venn diagram analysis of the overlapping results identified five downregulated miRNAs as potential regulators of FAP in lung cancer: hsa-miR-30b-5p, hsa-miR-624-5p, hsa-miR-30c-5p, hsa-miR-27b-3p, and hsa-miR-30a-5p (Figure 4B). Among these, miR-624-5p was selected for further investigation due to the lack of prior reports on its role in fibroblasts.

To investigate the expression level of miR-624-5p in tumors, we used the CancerMIRNome database and discovered that miR-624-5p was downregulated in several tumor tissues compared to normal tissues, including lung adenocarcinoma, lung squamous cell carcinoma, cholangiocarcinoma, and hepatocellular carcinoma (Figure 4C). We also utilized the E-MTAB-8026 database to compare the expression levels of miR-624-5p in the circulating blood of healthy individuals and lung cancer patients. The results found that miR-624-5p levels were significantly lower in the blood of lung cancer patients compared to healthy individuals (*p* < 0.001, Figure 4D).

To assess miR-624-5p expression in fibroblasts, we compared its levels between CAFs and NFs via qRT-PCR. The results revealed a significant downregulation of miR-624-5p in CAFs compared to NFs (*p* < 0.001, Figure 4E). We then modulated its expression by transfecting CAFs with a miR-624-5p mimic or NFs with an inhibitor. qRT-PCR confirmed successful overexpression in mimic-transfected CAFs and knockdown in inhibitor-transfected NFs compared to their respective negative controls (both *p* < 0.001, Figure 4F). Functional assays demonstrated that miR-624-5p overexpression significantly suppressed both proliferation and collagen contraction in CAFs (*p* < 0.01 and *p* = 0.002, respectively; Figure 4G,H). Conversely, its knockdown in NFs markedly enhanced these phenotypes (*p* < 0.01 and *p* < 0.001, respectively; Figure 4G,H). Collectively, these findings indicate that miR-624-5p is downregulated in CAFs and acts as an inhibitor of CAF activation.

### 3.5. Overexpression of miR-624-5p in CAFs Inhibited the Proliferation, Migration, and Invasion of NSCLC Cells

**Figure 5:**
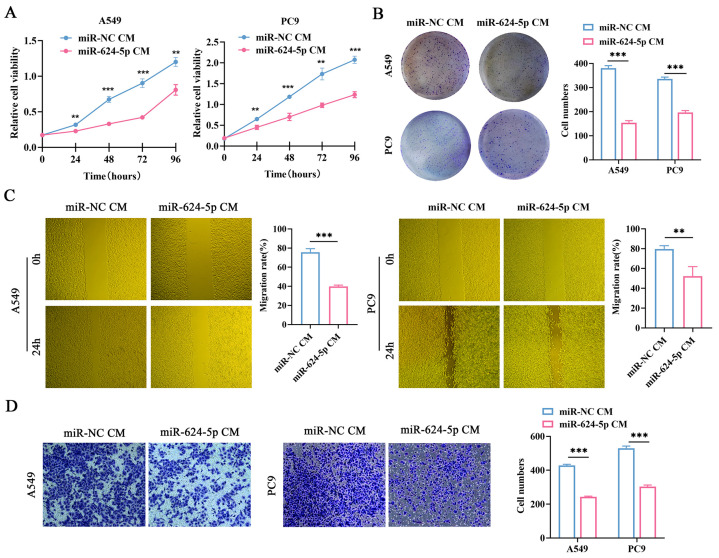
The effect of miR-624-5p in CAFs on the proliferation, migration, and invasion abilities of A549 and PC9 cells. (**A**) The proliferation viability of A549 and PC9 cells treated with miR-624-5p CM or miRNA-NC CM was evaluated by CCK-8 assay. (**B**) The colony formation ability of A549 and PC9 cells treated with miR-624-5p CM or miRNA-NC CM was evaluated by colony formation assay. (**C**) The wound healing assay to assess the effect of overexpressing miR-624-5p in CAFs on the migration ability of A549 and PC9 cells (×200). (**D**) Transwell co-culture assay to assess the effect of overexpressing miR-624-5p in CAFs on the invasion ability of A549 and PC9 cells, crystal violet staining (×200). ** *p* < 0.01, *** *p* < 0.001.

To investigate the effects of miR-624-5p in CAFs on the malignant behaviors of NSCLC cells, we conducted functional experiments. miR-624-5p mimic and miR-NC were transfected into CAFs, and the conditioned media (CM) were collected. Then, the A549 and PC9 cells were treated with fibroblast-derived CMs (miR-624-5p CM, miR-NC CM). CCK-8 assay and colony formation assay demonstrated that miR-624-5p CM obviously suppressed the proliferation and colony formation abilities of NSCLC cells (Figure 5A,B). The wound healing and transwell co-culture assays showed that compared to the miR-NC CM group, the miR-624-5p CM group significantly reduced the migration (*p* < 0.001, Figure 5C) and invasion capabilities (*p* < 0.001, Figure 5D) of A549 and PC9 cells. These findings suggest that overexpression of miR-624-5p in CAFs significantly inhibits the malignant behaviors of ESCC cells.

### 3.6. miR-624-5p Inhibits CAF Activation by Downregulating FAP

**Figure 6:**
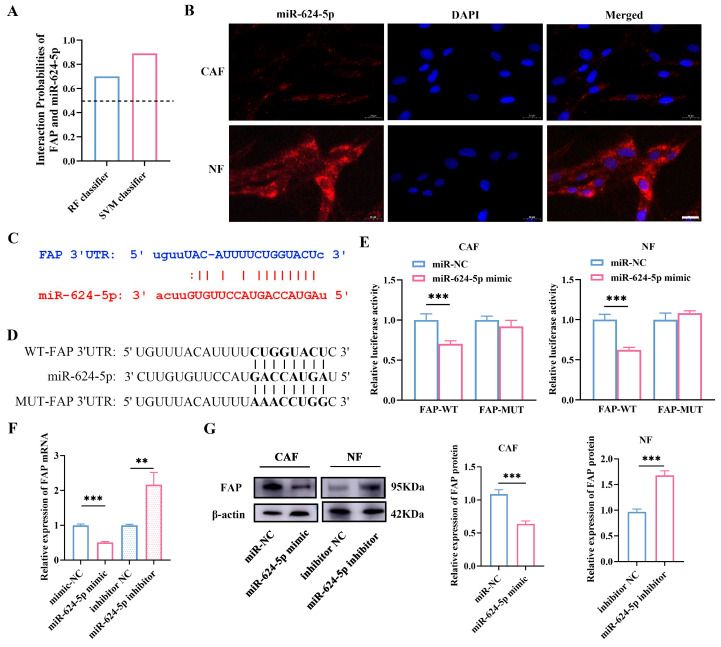
Prediction and validation of FAP as a target gene of miR-624-5p in CAFs. (**A**) Bioinformatics prediction of the potential binding probability of miR-624-5p and FAP. The interaction score > 0.5 was considered a close interaction. (**B**) FISH determined the subcellular localization of miR-624-5p. miR-624-5p was stained red. Nuclei (blue) were stained with DAPI (scale bar = 20 μm). (**C**) The predicted binding sites between miR-624-5p and the 3′UTR of FAP. (**D**) The sequences of WT-FAP 3′UTR and MUT-FAP 3′UTR. (**E**) Dual-luciferase reporter assay to detect the binding ability of miR-624-5p to FAP in CAFs and NFs. (**F**) The effect of miR-624-5p on the expression of FAP mRNA in fibroblast cells. (**G**) Western blotting analysis to assess the effect of miR-624-5p on FAP expression in CAFs and NFs. ** *p* < 0.01, *** *p* < 0.001.

To determine whether miR-624-5p inhibits CAF activation through FAP, we used the bioinformatic tool RPISeq to predict a potential binding between miR-624-5p and FAP (Figure 6A). FISH verified miR-624-5p was mainly located in the cytoplasm of fibroblast cells, suggesting that miR-624-5p might regulate expression of the target protein at the posttranscriptional level (Figure 6B). Additionally, we detected complementary binding sites between miR-624-5p and the 3′UTR of FAP using the TargetScan database, suggesting that FAP may be a target gene of miR-624-5p (Figure 6C). To confirm whether miR-624-5p targets FAP, we performed luciferase reporter assays with wild-type and mutant FAP (Figure 6D). The results showed that the miR-624-5p mimic significantly reduced the luciferase activity of the wild-type FAP, but did not affect mutant FAP in both CAFs and NFs (both *p* < 0.001, Figure 6E). Moreover, miR-624-5p mimic markedly decreased the expression levels of FAP mRNA in CAFs, whereas the inhibitor elevated it in NFs (*p* < 0.01, Figure 6F). Western blot analysis revealed that the expression of FAP protein was reduced in the miR-624-5p mimic group compared to the miR-NC group in CAFs (*p* < 0.001, Figure 6G, the uncropped scans of the Western blot images are provided in Figure S1). Conversely, the expression of FAP protein was elevated in the miR-624-5p inhibitor group relative to the inhibitor NC group in NFs (*p* < 0.001, Figure 6G). These results indicate that FAP is a downstream target gene of miR-624-5p, and miR-624-5p negatively regulates FAP expression, thus inhibiting CAF activation.

## 4. Discussion

Despite the advances in targeted therapies and immunotherapies, NSCLC poses a significant global health burden due to its high incidence and mortality rates [1]. The metastatic process in NSCLC, which is largely orchestrated by the dynamic tumor microenvironment (TME), remains a critical obstacle to improving therapeutic outcomes [24]. Within this complex network, CAFs play a pivotal role in driving metastatic progression and contributing to poor prognosis [25,26]. Therefore, elucidating the activation mechanisms of CAFs offers a promising avenue for developing precision treatments for NSCLC.

In this study, we assessed FAP expression in tumor lesions of NSCLC patients using ^68^Ga-FAPI-04 PET/CT imaging. FAP inhibitors (FAPIs) are small-molecule compounds designed on a quinoline scaffold. Upon radiolabeling—such as with ^68^Ga—they have been utilized as novel tracers for imaging malignant tumors [27,28]. Previous studies have noted that ^68^Ga-FAPI PET/CT may be superior to ^18^F-FDG PET/CT in the staging of lung cancer, especially in identifying distant metastases [29,30]. Moreover, Mona CE. et al. demonstrated a strong correlation between ^68^Ga-FAPi-46 PET biodistribution and FAP expression across multiple cancer types, underscoring the promise of FAPi PET as a pan-cancer biomarker for quantifying FAP expression [31]. Our results demonstrated higher FAP expression in primary tumors from advanced NSCLC cases compared to early-stage patients, supporting the hypothesis that FAP-expressing CAFs promote cancer metastasis, consistent with previous reports [32,33]. These findings clinically establish that FAP expression is associated with NSCLC metastasis and indicate that its molecular imaging signature can serve as a non-invasive biomarker for predicting metastatic progression. Importantly, this supports the growing perspective that FAPI-based imaging may be used not only for diagnosis and staging, but also to optimize patient selection for targeted therapies and to monitor treatment responses. Thus, our work provides a valuable translational foundation for further mechanistic investigation and for advancing image-guided strategies in NSCLC management.

To investigate the molecular mechanisms through which CAFs promote NSCLC metastasis, we isolated human primary CAFs and NFs in NSCLC and found that CAFs exhibit a stronger effect on NSCLC cell migration and invasion compared to NFs, providing insights into the interactions between CAFs and NSCLC cells. MiRNA dysregulation is intricately involved in the formation and functional programming of CAFs [34]. In our study, we identified miR-624-5p as a downregulated microRNA in CAFs in NSCLC. Overexpression of miR-624-5p led to decreased proliferation and collagen contraction capabilities of CAFs, indicating that miR-624-5p inhibits activation and function of CAFs. Additionally, the gain and loss-of-function experiments demonstrated that overexpressing miR-624-5p in CAFs significantly inhibited the proliferation, migration, and invasion abilities of A549 and PC9 cells, suggesting that the absence of miR-624-5p promotes CAF activation, thus facilitating NSCLC progression and metastasis.

Moreover, to elucidate the specific mechanisms through which miR-624-5p enhances NSCLC progression and metastasis, we initially investigated its localization in CAFs and NFs using the FISH assay. The results revealed that miR-624-5p predominantly localizes to the cytoplasm in fibroblast cells. We then utilized bioinformatics methods to predict the binding sites between miR-624-5p and the 3′UTR of FAP. Subsequently, dual-luciferase assay and Western blotting confirmed that miR-624-5p is an upstream target gene of FAP and miR-624-5p negatively regulates FAP expression. These results indicated that the loss of miR-624-5p in CAFs may be one of the mechanisms underlying the upregulation of FAP expression and maintenance of CAF activation. Our functional co-culture assays confirm that the miR-624-5p/FAP axis in CAFs critically regulates the pro-tumorigenic capacity of the secretome. Mechanistically, this is likely mediated through FAP-dependent pathways. In support, prior work shows that the circNOX4/FAP axis specifically drives IL-6 secretion in NSCLC [35], and FAP has been reported to regulate other key factors such as VEGF, CXCL12, and ECM-remodeling components [36,37]. Future studies will directly profile these downstream effectors to fully elucidate the secretory network controlled by this axis.

Our study identifies miR-624-5p as an upstream regulator of FAP in CAFs, highlighting FAP as a stromal target in NSCLC. Notably, dipeptidyl peptidase 4 (DPP4), another member of the serine protease family, exhibits a more complex role within the TME. DPP4 has been documented to function as both a tumor promoter and a suppressor across various cancers, affecting processes such as fibroblast activation and immune cell function [38,39,40]. This functional ambiguity, coupled with the broad tissue distribution of DPP4, limits its feasibility as a direct anticancer target [39]. In contrast, the restricted expression and consistent protumor function of FAP have facilitated its clinical translation, as evidenced by the clinical implementation of ^68^Ga-FAPI PET/CT imaging and ongoing trials of FAP-targeted radioligand therapies and antibody-drug conjugates [41,42]. Thus, by elucidating the miR-624-5p-mediated regulation of FAP, our work not only uncovers a novel mechanism of CAF activation but also proposes a biomarker and combination strategy to enhance FAP-targeted diagnostics and therapies.

While this study sheds light on the modulation of CAF activation, several limitations must be acknowledged. Firstly, the molecular mechanisms are largely drawn from in vitro experiments using patient-derived cells. Although these models are invaluable for their relevance to human biology, they cannot fully capture the complexity of stromal-tumor interactions in vivo. Therefore, the miR-624-5p/FAP axis requires further validation in vivo models. Furthermore, the heterogeneity of CAFs suggests that our findings are primarily applicable to the FAP-high subset, and the role of miR-624-5p across various CAF subsets requires further exploration. Secondly, the clinical correlation between FAP and tumor progression is based on a small, retrospective, single-center study, which may limit the generalizability of our findings. The prognostic and predictive value of the miR-624-5p/FAP axis should be established through prospective, large multi-center clinical studies with ^68^Ga-FAPI PET/CT imaging and associated survival outcomes in the future.

## 5. Conclusions

In summary, this study integrates ^68^Ga-FAPI-04 PET/CT imaging with molecular mechanistic analysis to clarify a key pathway underlying metastatic progression in NSCLC. We demonstrate that elevated FAP expression is associated with cancer metastasis and identify miR-624-5p as an upstream regulator that suppresses FAP expression and CAF activation, thereby inhibiting NSCLC metastasis. Future studies should focus on validating the miR-624-5p/FAP axis in expanded patient populations and preclinical models, as well as exploring integrated CAF-targeted and imaging-guided therapeutic strategies in NSCLC.

## Figures and Tables

**Table 1:** Clinicopathological characteristics of NSCLC patients (*n* = 61).

Clinicopathological Parameter	Number of Cases (Proportion)
Sex	
Male	36 (59.0%)
Female	25 (41.0%)
Age at diagnosis	
<60	13 (21.3%)
≥60	48 (78.7%)
Smoking history	
Yes	26 (42.6%)
No	35 (53.4%)
Histological type	
Adenocacinoma	41 (67.2%)
Squamous cell carcinoma	15 (24.6%)
Others	5 (8.2%)
TNM Stage	
I	18 (29.6%)
II	11 (18.0%)
III	24 (39.3%)
IV	8 (13.1%)

## Data Availability

The clinical data in this study are available from the corresponding authors upon reasonable request. The datasets used in this study are available from the following sources: GEO datasets (GSE169587, https://www.ncbi.nlm.nih.gov/geo, (accessed on 5 August 2024)); Targetscan (http://www.Targetscan.org, (accessed on 10 August 2024)); DIANA-microT-CDS (http://diana.imis.athena-innovation.gr/DianaTools/index.php?r=microT_CDS/index, (accessed on 10 August 2024) ); miRmap (https://mirmap.ezlab.org/, (accessed on 10 August 2024)); CancerMIRNome database (http://bioinfo.jialab-ucr.org/CancerMIRNome/, (accessed on 6 August 2024)); E-MTAB-8026 database (https://www.ebi.ac.uk/biostudies/arrayexpress, (accessed on 6 August 2024)); RPISeq (http://pridb.gdcb.iastate.edu/RPISeq/about.php, (accessed on 3 November 2024)).

## Supplementary Materials

The following supporting information can be downloaded at: https://www.mdpi.com/article/10.3390/cancers18020279/s1, Figure S1: Uncropped Western blots corresponding to Figure 6G.

## Abbreviations

The following abbreviations are used in this manuscript:

CAFs	Cancer-associated fibroblasts
NFs	Normal fibroblasts
NSCLC	Non-small cell lung cancer
TME	Tumor microenvironment
FAP	Fibroblast activation protein
FAPI	Fibroblast activation protein inhibitor
PET	Positron emission tomography
MIP	Maximum intensity projection
SUVmax	Maximum standardized uptake value
SUVmean	Mean standardized uptake value
MiRNA	MicroRNA
qRT-PCR	Real-time quantitative polymerase chain reaction
FISH	Fluorescence in situ hybridization
CAF-CM	CAF-derived conditioned medium
NF-CM	NF-derived conditioned medium
UTR	Untranslated region
