# Supplementary figures and images for: Modulation of Cancer-Associated Fibroblasts via the miR-624-5p/FAP Axis Drives Progression and Metastasis in Non-Small Cell Lung Cancer
Source: Cancers (Basel). 2026 Jan 16;18(2):279. doi: 10.3390/cancers18020279 (PMC12839361; doi:10.3390/cancers18020279)

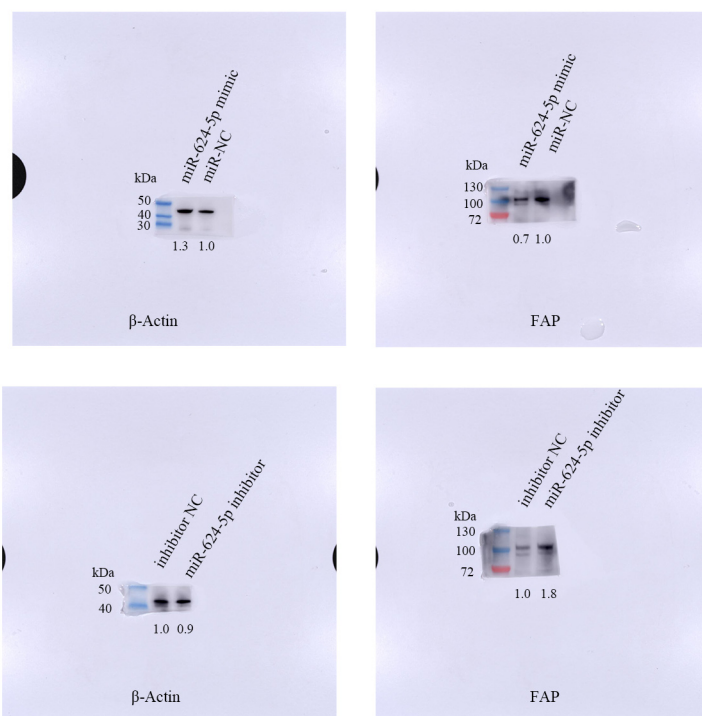

**Figure S1.** Uncropped Western blots corresponding to Figure 6G.

Supplement: Supplementary file 1 [file cancers-18-00279-s001.zip › cancers-4031957-supplementary.pdf]
